# Editorial: New Insights on Seminal Factors Signaling Female Reproduction in Mammals

**DOI:** 10.3389/fvets.2022.893379

**Published:** 2022-05-19

**Authors:** Margherita Maranesi, Massimo Zerani, Mauricio Silva

**Affiliations:** ^1^Department of Veterinary Medicine, University of Perugia, Perugia, Italy; ^2^Department of Veterinary Science and Public Health, Catholic University of Temuco, Temuco, Chile

**Keywords:** nerve growth factor, β-NGF, seminal plasma, ovulation, ovary, corpus luteum

This Research Topic aimed to increase our understanding of the effects of seminal factors on female reproduction in mammals.

These seminal factors, produced in the male genital system, can exert their action in the female, intervening in many reproductive processes. For this reason, the study of these molecules of male origin in the female helps us to broaden our knowledge on their action in the female. Therefore, another aim of this Research Topic was to study the role of these factors with respect to luteal and follicular activity.

Although significant advances have been made regarding our knowledge of the control of female reproduction, there are still several aspects which have not been deeply studied. One of such areas is the influence that factors present in the seminal fluid (i.e., proteins, growth factors, interleukins, etc.) can exert once they are deposited in the female reproductive tract during ejaculation. Several reproductive phenomena are potentially influenced or modified by seminal factors of different chemical nature. In the last decades one of the most studied seminal factors is nerve growth factor β (β-NGF) (1–3). This protein factor present in high concentration in the semen of several induced ovulation species can effectively trigger ovulation. However, besides β-NGF several other seminal signals modulate female reproduction in many mammalian species which study warrants further investigation.

This special e-collection includes four papers covering the above mentioned aspects.

In their review Robertson and Martin, analyze the effects of seminal fluid on female reproduction far beyond ovulation. Interestingly, the authors assert that the components of seminal fluid meet the classic definition of pheromone (4), and therefore should be considered as such. Furthermore, these components of male origin (transforming growth factor-β, 19-OH prostaglandins, various ligands of Toll-like receptor-4, and cyclic ADP ribose hydrolase) (5), would act in the female not only affecting the gonadal axis, but also modulating its immune system, affecting reproductive success. Seminal factors sensitize the female genital tract after ejaculation priming the female immune response, conferring activated T-cell tolerance and allowing the presence of histocompatibility antigens inherited from the male into the conceptus, finally promoting uterine receptivity and embryo implantation (6).

This interesting process sees the important consequence that this male-female interchange translates into the possibility of a transmission of that male germline. In this case a “cryptic female choice” is generated, a process through which the females interrogate the reproductive potential of the hypothetical male partners and decide to invest or not reproductive resources (7). Resources invested from the cryptical female choice contribute at the promotion of successful fertilization and at the adaptative evolution of male accessory glands protein secretion (8), helping to ensure optimal female reproductive resource investment and maximal progeny fitness (9).

Stewart et al. present striking effects of bovine seminal NGF on steroidogenesis and angiogenic markers in the bovine pre-ovulatory follicle. The authors state that bull seminal plasma contains relevant concentrations of NGF, particularly into the sperm-rich fraction of the ejaculate (10). Seminal NGF would be transported to the ovary through the local counter-current exchange between the uterine venous and the ovarian artery that allows prostaglandin F2α (PGF2α) direct transport from the uterus to the ovary during luteolysis (11). Recombinant NGF *in vitro* increased androstenedione and progesterone release, PGE production, and cell proliferation in bovine (4). Using *in vitro* culture of bovine granulosa and theca cells, Stewart et al. reported that seminal NGF generated up-regulating effects on gene expression of steroidogenic enzyme HDS17B and testosterone production, whereas gene expression of the angiogenic enzyme FGF2 was downregulated. Instead, progesterone and estradiol productions and PGES, VEGFA, and ESR1 activities were not modify by NGF treatment. The authors concluded that seminal plasma NGF directly affect theca and granulosa cells of the bovine pre-ovulatory follicle, stimulating testosterone production and cell proliferation, with a hastened onset of follicle wall cellular remodeling induced by FGF2 expression decreasing.

Another aim of the special issue was to provide new information on the development and regression of the corpus luteum and ovarian follicles.

The luteal gland undergoes a continuous formation and regression processes under the action of various luteotrophic or luteolytic factors (12, 13). On this regard, in this special issue Jonczyk et al. expanded the knowledge in the field of the acquisition of luteolytic competence in the bovine corpus luteum, by injecting both intramuscularly and intraluteally an analog of PGF2α: dinoprost. Their study demonstrated that intra-CL injection of dinoprost increases oxytocin concentrations and decreases intravenous progesterone (P4) levels, in a dose-dependent manner, in cows at the mid-luteal phase. An increase in indicators of vascularization of CL (CL blood flow and adjusted corpus luteum blood flow), accompanied by a drop in P4 level, was observed 2 h after intra-CL dinoprost injection in the middle-stage CL. Moreover, the lack of changes in blood flow and P4 concentration at the early luteal phase of the estrous cycle appeared to be directly correlated with the resistance of CL to the action of dinoprost injected directly into the early-stage CL. Furthermore, the decrease in STAR mRNA and increases in receptor-interacting protein kinases 1 (RIPK1) and receptor-interacting protein kinases 3 (RIPK3) mRNA levels confirmed that 2.5 mg of dinoprost injected directly into CL is a minimum dose that will induce the luteolytic cascade. This study opens up new possibilities for the use of intra-corpus luteum application of drugs/hormones that could be a prevalent tool in bovine reproduction, from a therapeutic and synchronization aids.

Lastly, in their present study Yu et al. addressed the possible interaction and synergy between anti-Müllerian hormone (AMH) and inhibin (INH) in modulating primary granulosa cells steroidogenesis and, thus, their possible effect on mice fertility. Both, INH and AMH belong to transforming growth factor β (TGF-β) superfamily. INH regulates ovarian functions by suppressing FSH secretions through pituitary–gonadal negative feedback (14). In theca cells, INH affects StAR and HSD3B expression and androgen production (15), whereas, its role in granulosa cells steroidogenesis is ambiguous (16). AMH plays an important role in the sexual differentiation and gonadal function (17) participating in primordial follicle recruitment inhibition and pre-antral and antral follicle growth through the FSH regulation (18). In addition, FSH-dependent aromatase and estradiol production is abolished by AMH (19).

Using an *in vitro* granulosa cell culture system, Yu et al. demonstrates that both INH and AMH alone significantly attenuated FSH-induced steroid hormone secretion. In addition, INH and AMH together increased the inhibitory effect on FSH-induced estradiol and progesterone production, with a concomitant downregulation of FSH-stimulated CYP19A1, HSD3B, CYP11A1, StAR expression. Utilizing female mice immunized against INH and AMH eukaryotic expression plasmids, these authors observed that co-immunization increased estradiol levels, resulting a more litter size, but not an offspring's weight. The authors concluded that steroidogenesis and the litter size in mice is modulated by INH and AMH in a synergistic way.

Our knowledge of the control of female and male reproduction is ever evolving. Interestingly, the classic approach has been to study them separately, as if no connection between them existed. The growing notion that mating is not just a mere mechanism for the male to deposit its sperm into the female reproductive tract, but rather a sort of chemical communication between both parts involved, with relevant consequences such as the success of pregnancy, is enlarging our views of how we understand courtship and mating behavior, and value its long-term consequences.

During the last decade proteomic studies of seminal fluid in several animal species have opened a wide window of knowledge, showing the effects that many male-derived factors have on the control of female reproduction. In this sense, the proposition of Robertson and Martin to enlarge the classic concept of pheromones including chemical factors present in semen should be taken into consideration, since recognizes and validate this newly discovered chemical communication between male and female. Special attention should be given to the presence of β-NGF in the seminal fluid of induced and spontaneous ovulators, since as demonstrated in camelids and rabbits, its presence and potent ovulation-induction effect could account for a new category of induced ovulators, where seminal chemical signs control, completely or partially, the occurrence of ovulation.

Undoubtedly the use of *in vitro* and *in vivo* models to evaluate the effects of several of these seminal components on female reproduction (ovulation, corpus luteum function, embryo survival, and pregnancy) is a valuable tool that will continue to bring light into this fascinating field of study.

## Author Contributions

All authors listed have made a substantial, direct, and intellectual contribution to the work and approved it for publication.

## Conflict of Interest

The authors declare that the research was conducted in the absence of any commercial or financial relationships that could be construed as a potential conflict of interest.

## Publisher's Note

All claims expressed in this article are solely those of the authors and do not necessarily represent those of their affiliated organizations, or those of the publisher, the editors and the reviewers. Any product that may be evaluated in this article, or claim that may be made by its manufacturer, is not guaranteed or endorsed by the publisher.
